# The Record of the Year

**Published:** 1897-12

**Authors:** 


### Editorial.

### THE RECORD OF THE YEAR.

The good business man at the close of the year takes account of stock, estimates profits and loss, and arranges for the future. A profession should in the same way and for similar reasons devote a portion of its time at the same period to a summing up of its gains and losses during the past twelve months, that it may treasure the one and avoid the mistakes that led to the other.

In some degree the past months have made history which may leave its mark on the future, it is hoped for good, and, on the other hand, it is thought something has been lost.

The close of the year finds dentistry in the United States in its organized capacity in somewhat of a chaotic condition ; and while the pessimistic state of mind is very rarely, perhaps, the best, it is impossible to look forward to the coming year of 1898 with the same feeling of confidence that opened the year 1897.

The dental profession at that time looked forward with satisfaction to a year of positive advancement in organized effort. The various bodies, both State and local, gave the subject of reorganization special consideration. It was discussed, as never before, publicly and privately, with the result that the final action at Old Point Comfort in August left the matter practically as it was prior to that event.

This subject has been considered in a previous article, and it is mentioned here simply as an illustration of the active thought at the beginning of the year. There has been reorganization. The American Dental Association was permitted to die and the Southern to live. A partial union of the associations North and South was effected, and dentists everywhere are promised that this will result in all the good anticipated. At present the " wish seems father to the thought," for the signs in the professional heavens do not seem propitious.

When January, 1897, opened the year, harmony reigned in college circles. The National Association of Dental Faculties held out the hand of good-fellowship to the National Association of Dental Examiners, notwithstanding many disagreeable antagonisms. That body permitted itself to be dominated by bad advisers, with the result that the close of the year finds the dental colleges of the United States in direct antagonism to that organization and almost in open war against laws regulating practice in general. That this is to be lamented requires no word from this journal, but that it is to be wholly regretted cannot be written, for it is through violent ebullitions that an equilibrium is established in the mental as well as in the physical world. Hence these disturbed relations are not regarded as an unmixed evil, but rather a necessary condition prior to that period which will surely come, when all conflicting interests will be arranged upon a satisfactory basis.

The progress professionally has not been marked during the year. While there has been activity and some noted contributions to the literature of the profession, there has been no special work that distinguishes this year from those that preceded it. This was to be expected, for dentistry has its limitations, and while it is not " written up," as expressed by another, it is vain to anticipate any great revelations in the near future. We, as dentists, must labor on, perfecting the old lines and accepting the new as they appear in the progress of events.

The most encouraging feature of the past year has been seen in dental educational circles. This to some may seem to be an absurd statement in view of the fact of the action of the National Association of Dental Faculties at Old Point Comfort; but it is nevertheless true that college work and the real standard of that body, and those associated with it, has made marked advance in the past twelve months. The writer of this has, perhaps, a quite intimate knowledge of the work of the schools, and he feels convinced that at no period has there been a more earnest desire to eliminate all that is defective than at the present time. There was nothing of a retrograde character at Old Point, nor has there ever been in that Association but one feeling,—that the schools must progress as fast as circumstances would permit. There is a feeling abroad that there are too many schools. This may be true, but it is doubted, and the cry of commercialism is heard everywhere. While this to some extent is justified, it must be remembered that nothing involving financial outlay can be carried on successfully without business methods. It should also be considered that the majority of the smaller colleges are kept alive through great sacrifices on the part of the faculties, and the huge emoluments so often and so offensively stated are, in the main, pure myths. It is a gratification, therefore, that men in the past twelve months have been found willing to make sacrifices for the good of their calling by engaging in a labor the only returns of which can be found in the satisfaction of helping along educational work. The attitude of many towards these educational labors has no justification, and constitutes one of the dark spots on the history of the passing months.

One of the most pleasing features for the present and the future is that the past year has given the dental profession an objective lesson in college work. At no period has there been greater efforts made to erect structures worthy of the labor to be performed in these training institutions. There is nothing which so gauges the real status of a profession as its buildings and appliances for teaching. Judged by this standard, the dental profession has grown the past year as in no period antedating this. Some of the colleges have erected large and architecturally beautiful structures, and with every convenience, not only for the teaching of dentistry proper but of all the collateral branches. That this has been only possible through a very large outlay of money need not be stated. Men do not invest from a hundred to a hundred and fifty thousand dollars in a building on mere sentiment, and yet sentiment, or a desire to meet the demands of the period, is the sole actuating motive. When critics carp at the non-progressive character of colleges they may compare these fine structures with the early beginning and then consider the question, Have colleges, and through colleges the dental profession, gained not only in the past year but in the past century ?

There is, therefore, no need of discouragement. The disturbed condition of the present presages the period of calm. Better by far frictional activity than the dead level of inertia.

The past is behind us, the present is ours, and the future of possibilities lies before us. He is wise who accepts all and labors patiently, well assured that amid it all progress is the eternal condition of mentality, and the apparent lapses are intrinsically but the outward signs of that activity that leads forever to a higher standard.

